# Reciprocal relationships between adolescent mental health difficulties and alcohol consumption

**DOI:** 10.1007/s00787-025-02644-6

**Published:** 2025-01-18

**Authors:** Janet Kiri, James Hall, Samuele Cortese, Valerie Brandt

**Affiliations:** 1 School of Psychology, Centre for Innovation in Mental Health, University of Southampton, University Road, Southampton, SO17 1BJ UK; 2 Southampton Education School, University of Southampton, Southampton, UK; 3 Clinical and Experimental Sciences (CNS and Psychiatry), Faculty of Medicine, University of Southampton, Southampton, UK; 4 Solent NHS Trust, Southampton, UK; 5 Hassenfeld Children’s Hospital at NYU Langone, New York University Child Study Center, New York City, NY USA; 6 Division of Psychiatry and Applied Psychology, School of Medicine, University of Nottingham, Nottingham, UK; 7 DiMePRe-J-Department of Precision and Regenerative Medicine-Jonic Area, University of Bari “Aldo Moro”, Bari, Italy; 8 Clinic of Psychiatry, Social Psychiatry and Psychotherapy, Hannover Medical School, Hanover, Germany

**Keywords:** Developmental psychopathology, Alcohol consumption, Mental health difficulties, Random-intercept cross lagged panel model

## Abstract

The directionality of the relationship between adolescent alcohol consumption and mental health difficulties remains poorly understood. This study investigates the longitudinal relationship between alcohol use frequency, internalizing and externalizing symptoms from the ages of 11 to 17. We conducted a random-intercept cross-lagged panel model across three timepoints (ages: 11yrs, 14yrs, 17yrs; 50.4% female) in the Millennium Cohort Study (*N* = 10,647). Survey weights were used to account for attrition. At each timepoint, past month alcohol use frequency was self-reported, parents and cohort members reported internalizing/externalizing symptoms using the Strengths and Difficulties Questionnaire. We controlled for alcohol expectancies, sex, and four cumulative risk indices (perinatal risk, early childhood adverse parenting, longitudinal parent-level risk occurrence, and persistent household socioeconomic deprivation). More frequent past month alcohol use at age 11 predicted increased internalizing symptoms at age 14 (β = 0.06; *p* =.01). More frequent past month alcohol use at age 14 predicted increased externalizing symptoms at age 17 (β = 0.11; *p* <.001). Increased internalizing symptoms consistently predicted reduced alcohol use at the next timepoint throughout the study period (11 years: β= -0.04; *p* =.03; 14 years: β= -0.09; *p* <.001). Increased externalizing symptoms at age 11 predicted increased alcohol consumption at age 14 (β = 0.06; *p* =.004). Frequent adolescent alcohol consumption represents a significant risk for subsequent mental health difficulties. Externalizing symptoms and alcohol use frequency appear to exacerbate one another. Internalizing symptoms may reduce the risk of frequent alcohol consumption. Incorporating routine alcohol screening into adolescent mental health treatment settings could reduce the risk of comorbid externalizing and alcohol use disorders.

**Supplementary Information:**

The online version contains supplementary material available at 10.1007/s00787-025-02644-6.

## Introduction

Adolescents are particularly prone to engaging in risky behaviours [1]. For instance, in the UK around 50% of adolescents report experimenting with alcohol, drugs, or tobacco by the age of 14 [2]. Adolescence also comprises a key developmental period for the emergence of several psychiatric disorders [3], with nearly half of individuals worldwide reporting the onset of symptoms before age 18 [4].

Adolescent alcohol consumption and mental health difficulties are intimately related [5]. Frequent adolescent alcohol consumption represents a risk factor for the development of Alcohol Use Disorders (AUDs) and various psychiatric disorders in adulthood [6–8]. Similarly, adolescent mental health difficulties have been found to reflect robust risk factors for a range of psychiatric disorders and AUDs in adulthood [9], suggesting that mental health difficulties and frequent alcohol consumption during adolescence may place individuals at a heightened risk of developing comorbid AUDs and psychiatric disorders later in development [10]. Comorbidity between AUDs and psychiatric disorders, compared to either in isolation, has been linked to more severe symptomatology and functional impairment [11].

However, it remains unclear how alcohol use and mental health difficulties influence each other before adulthood [10]. The few studies that have explicitly investigated the reciprocal relationship between alcohol consumption and mental health difficulties in adolescence have produced mixed findings [12–14]. Furthermore, previous research investigating the temporal sequencing of co-occurring AUDs and psychiatric disorders has often relied on retrospective reports collected after the diagnosis of one or both conditions [10, 15]. Several risk factors, such as prenatal alcohol exposure, negative parenting styles, poor parental mental health, and socioeconomic deprivation, have been implicated in the development of psychiatric disorders and AUDs [16–18]. However, prior longitudinal studies examining the relationship between mental health difficulties and alcohol consumption have either controlled for a limited number of risk factors collected at a single timepoint [14] or have investigated risk factors from a single domain [12]. Clarifying the nature of the relationship between mental health difficulties and alcohol consumption would better inform preventative efforts that could be implemented starting in early adolescence.

Moreover, prospective longitudinal investigations using statistical approaches that separate the stable trait-like differences across individuals (between-person associations) from an individual’s fluctuations in alcohol consumption and reported mental health difficulties over time (within-person associations), prior to the emergence of AUDs, may provide insight into the developmental pathways to comorbid AUDs and psychiatric disorders in adulthood. Whilst employing approaches that disassociate within-person from between-person effects does not provide an absolute indication of causality, it facilitates a better understanding of the temporal predominance between mental health difficulties and alcohol consumption during adolescence [19, 20].

The current study examined whether there is a reciprocal relationship between mental health difficulties and alcohol consumption from the ages of 11 to 17, dissociating within-person from between-person associations, and controlling for shared risk factors at the perinatal, parent, and household level [21]. Using a random-intercept cross-lagged panel model (RI-CLPM), we aimed to clarify the temporal sequencing and directionality of the relationship between mental health difficulties and monthly alcohol use frequency. We hypothesised that there would be significant reciprocal relationships, whereby increases in reported mental health difficulties would precede increases in alcohol consumption (and vice versa) across the study period.

The current study used data from the British Millennium Cohort Study (MCS) which follows a sample of around *N* = 19,500 children (and their families) since their birth in 2000–2001. Detailed data collection, sampling and stratification procedures have been described elsewhere [22]. There were seven waves of data collection: at 9 months (T1), 3 years (T2), 5 years (T3), 7 years (T4), 11 years (T5), 14 years (T6), and 17 years (T7). Information was collected on a range of topics including mental health, finances, and parent-child relationships. Parents provided written informed consent at each timepoint for the participation of them and their child and for the data to be made available for secondary data analysis through the UK Data Archive: https://www.data-archive.ac.uk/. Ethical approval for this secondary data analysis was granted by the University of Southampton ethics committee (ERGO: 79894.A1).

## Methods

### Participants

The final analytical sample comprised *N* = 10,647 participants (50.4% female; 79.2% Caucasian). Inclusion/exclusion criteria are described in detail in Supplementary Figure S1. Participants who did not complete any alcohol or mental health measures or lacked survey weights at T7 were excluded from the analytical sample. In line with previous investigations of substance use in the MCS [23], participants who reported use of the fake drug “Semeron” at T7, were excluded.

### Primary measures

Alcohol use frequency was self-reported for the previous 30 days at each timepoint on the scale (0) “Never”, “, (1) “1–2 times”, (2) “3–5 times”, (3) “6–9 times”, (4) “10–19 times”, (5) “20–39 times”, and (6) “40 or more times*”*. Due to the low volume of responses in some categories, responses were condensed into three categories: (0) “Never”, (1) “1–2 times per month”, and (2) “more than 3 times a month” [24]. Higher scores reflect more frequent monthly alcohol consumption.

Internalizing and externalizing symptoms were assessed by parent- and self-report, using the Strengths and Difficulties Questionnaire (SDQ) at each timepoint. The parent-report was used at ages 11 and 14, whereas the self-report was used at age 17 (see Supplementary Table S1 for further details). The SDQ has demonstrated clinical utility for predicting psychiatric disorders in normative samples [25]. For our purposes, internalizing symptoms were measured using the emotional problems subscale (range; 0–10), externalizing symptoms were measured with the hyperactivity/inattention and conduct problems subscales (range: 0–20). Higher scores indicate a greater number of symptoms.

### Covariates

To evaluate the influence of multiple risks that have been implicated in the development of AUDs and mental health difficulties, salient risk factors collected throughout the cohort member’s childhood were divided by ecological level (child, parent and household). The combined risk at each level was assessed via cumulative risk indices (CRIs) that accounted for the developmental timing of risk exposure (for detailed variable information see Supplementary Tables S1 and S2). Individual risks were dichotomized to reflect whether the cohort member reported (0) “no risk exposure” or (1) “risk exposure”, with CRI scores reflecting the number of risks encountered. CRIs involving risks collected across multiple timepoints were computed for participants with data on over 50% of respective indicators across multiple waves, while CRIs compiled of variables assessed at a single timepoint were computed for those with data on over 25% of respective indicators [26]. CRIs included the following: perinatal CRI, early childhood (EC) adverse parenting, longitudinal parent-level risk occurrence, and persistent household socioeconomic deprivation (SED). Additional details for the variables included in each CRI, alongside the computation of each CRI, are available in the Supplementary Material (see Supplementary Tables S1-S2). Self-reported alcohol expectancies at age 11, which have been implicated in the development of problematic alcohol use behaviours during adolescence and early adulthood [27], and the participant’s parent-reported biological sex were also controlled for (see Supplementary Table S1 for further details).

Table 1 presents an overview of the characteristics and differences between participants with at least one missing value on mental health difficulties or alcohol use, compared to participants with complete data across the three timepoints. There were significant differences between the complete and missing samples on all variables - except for monthly alcohol use at age 11 and internalizing symptoms at age 17 - with small to modest effect sizes (Cramer’s V ≤ 0.16, Cohen’s d ≤ 0.41). A full correlation matrix is presented in Supplementary Table S3.

**Table 1:** Characteristics and differences between sample with complete data on mental health difficulties and monthly alcohol use (*n* = 7172) and sample with at least one missing value (*n* = 3475)

	Complete data (*n* = 7172)			Missing data (*n* = 3475)				
	*N* (%)	Mean (SD)		*N* (%)	Mean (SD)	Chi-square (df)	*P* value	Effect size
Sex						48.23 (1)	< 0.001	0.07^b^
Male	3403 (34.6%)	-		1476 (15.0%)	-	-	-	-
Female	3769 (38.3%)	-		1192 (12.1%)	-	-	-	-
Ethnicity						242.07 (5)	< 0.001	0.16^b^
White	5901 (61.6%)	-		1695 (17.7%)	-	-	-	-
Mixed	323 (3.4%)	-		128 (1.3%)	-	-	-	-
Black	193 (2.0%)	-		118 (1.2%)	-	-	-	-
Indian	169 (1.8%)	-		103 (1.1%)	-	-	-	-
Pakistan	421 (4.4%)	-		307 (3.2%)	-	-	-	-
Other	125 (1.3%)	-		103 (1.1%)	-	-	-	-
Monthly alcohol use								
Age 11						1.02 (2)	0.60	0.01^b^
Never	6958 (71.6%)	-		2464 (25.3%)	-	-	-	-
1–2 times	182 (1.9%)	-		69 (0.7%)	-	-	-	-
3 or more times	32 (0.3%)	-		15 (0.2%)	-	-	-	-
Age 14						16.62 (2)	< 0.001	0.04^b^
Never	5611 (60.9%)	-		1681 (18.2%)	-	-	-	-
1–2 times	1143 (12.4%)	-		257 (2.8%)	-	-	-	-
3 or more times	418 (4.5%)	-		104 (1.1%)	-	-	-	-
Age 17						167.13 (2)	< 0.001	0.13^b^
Never	2553 (26.4%)	-		1249 (12.9%)	-	-	-	-
1–2 times	2310 (23.9%)	-		697 (7.2%)	-	-	-	-
3 or more times	2309 (23.9%)	-		562 (5.8%)	-	-	-	-
Internalizing symptoms								
Age 11	7172 (75.1%)	1.73 (1.90)		2384 (24.9%)	2.04 (2.13)	6.25 (3727)^a^	< 0.001	0.16^c^
Age 14	7172 (75.1%)	1.92 (2.08)		2379 (24.9%)	2.32 (2.24)	7.77 (3833)^a^	< 0.001	0.19^c^
Age 17	7172 (72.9%)	3.51 (2.45)		2665 (27.1%)	3.43 (2.46)	1.45 (9835)^a^	0.15	0.03^c^
Externalizing symptoms								
Age 11	7172 (76.8%)	3.98 (3.29)		2161 (23.2%)	5.09 (3.84)	12.19 (3173)^a^	< 0.001	0.33^c^
Age 14	7172 (75.2%)	3.92 (3.31)		2370 (24.8%)	5.32 (3.86)	15.88 (3586)^a^	< 0.001	0.41^c^
Age 17	7172 (72.9%)	5.54 (3.27)		2664 (27.1%)	5.81 (3.35)	3.57 (9834)^a^	< 0.001	0.08^c^

^a^Independent Samples t-test. ^b^Cramer’s V. ^c^Cohen’s d

### Statistical analysis

The analysis was conducted the “lavaan” package in the R environment [28].

A random-intercept cross-lagged panel model (RI-CLPM) [19] was employed to explore the dynamic relationship between monthly alcohol use, internalizing, and externalizing symptoms across T5-T7, controlling for sex, alcohol expectancies and salient cumulative risk factors. RI-CLPM demonstrates significant advantages over the traditional cross-lagged panel models used in previous research [12–14], through including random intercepts which enables the delineation of between-person from within-person variances [19]. Additional details regarding the RI-CLPM are available in the Supplementary Materials (see Supplement 1).

**Figure. 1:**
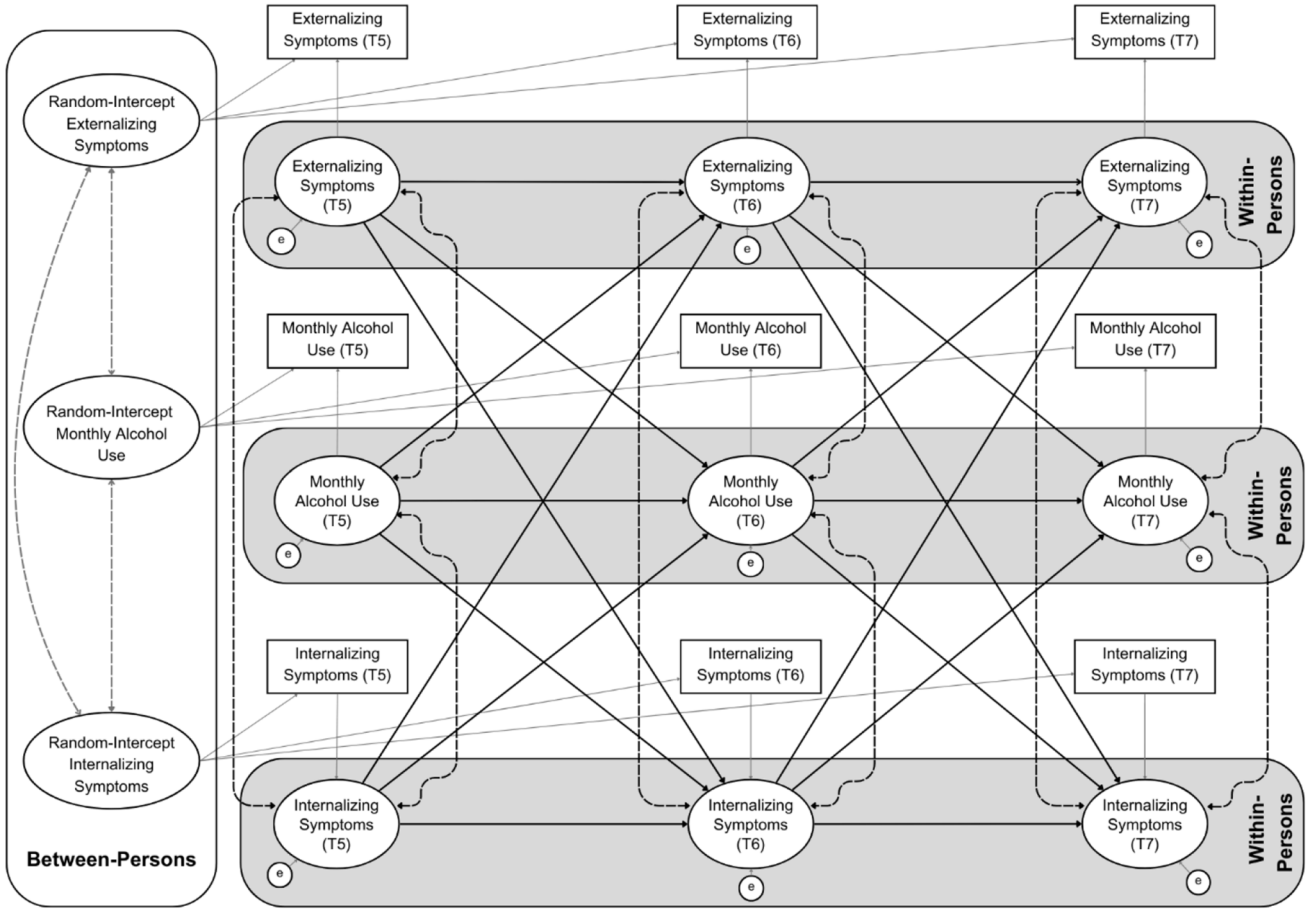
Conceptual diagram of the trivariate random-intercept cross-lagged panel model. T5: timepoint five (same pattern for subsequent timepoints). Cross-lagged and autoregressive paths (solid black lines), contemporaneous correlations at each timepoint (dashed black lines), between-person associations between the random-intercepts (dashed grey lines) are shown. All other paths are represented with solid grey lines for ease of interpretation

A trivariate (monthly alcohol use, internalizing and externalizing symptoms) RI-CLPM was conducted to adjust for the high co-occurrence between internalizing and externalizing symptoms. This approach inherently accounts for the shared variance between monthly alcohol consumption, internalizing, and externalizing symptoms at both the between-person and within-person level, enabling an exploration of the dynamic associations between these constructs throughout adolescence. Figure 1 present a conceptual diagram of the unconditional trivariate model [29]. The following predefined thresholds were used to assess model fit; Comparative Fit Index (CFI; good fit > = 0.95), Tucker-Lewis Index (TLI; acceptable fit > = 0.90, good fit > = 0.95), Standardized Root Mean Square Residual (SRMR; good fit < = 0.08) and Root Mean Square Error of Approximation (RMSEA; good fit < 0.05) [30].

Contemporary bivariate correlations among alcohol use, externalizing, and internalizing symptoms were estimated. Attrition and the clustered sampling design of the MCS sample were addressed with survey weights. Full Information Maximum Likelihood handled missing data, and due to skewed variables, Maximum Likelihood Estimation with Robust Standard Errors (MLR) was employed.

Conceptual Diagram of the Trivariate Random-Intercept Cross-Lagged Panel Model. T5: timepoint five (same pattern for subsequent timepoints). Cross-lagged and autoregressive paths (solid black lines), contemporaneous correlations at each timepoint (dashed black lines), between-person associations between the random-intercepts (dashed grey lines) are shown. All other paths are represented with solid grey lines for ease of interpretation.

## Results

### Longitudinal relationship between monthly alcohol use, internalizing and externalizing symptoms

The RI-CLPM model exhibited good fit (χ2(31) = 306.91, *P* <.001; RMSEA = 0.045 [0.04–0.05]; CFI = 0.97; TLI = 0.90; SRMR = 0.04).

**Figure. 2:**
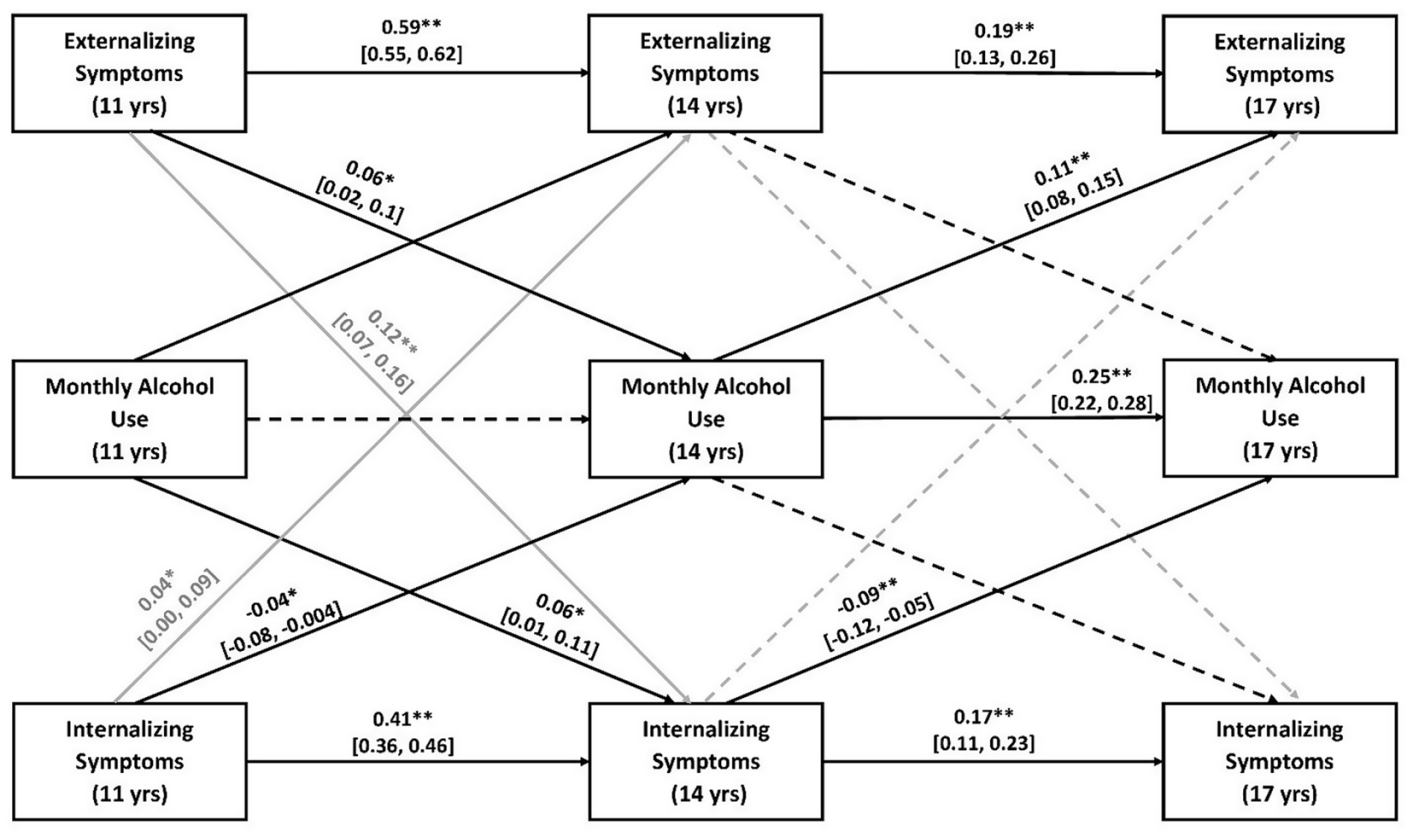
The cross-lagged and autoregressive paths between alcohol Use and internalizing/externalizing symptoms. This figure shows the paths (*p<.05, **p<.001), between monthly alcohol use frequency and internalizing/externalizing symptoms (presented in black) and between internalizing and externalizing symptoms (presented in grey). Solid lines represent significant paths. Dashed lines represent non-significant paths. Standardized estimates with 95% confidence intervals are presented for significant paths only

Increased internalizing symptoms at age 11 predicted reduced alcohol use at the next timepoint (β=-0.04; *SE =* 0.02; 95% CI, -0.08 to -0.004; *P* =.03), as did increased symptoms at age 14 (β=-0.09; *SE =* 0.02; 95% CI, -0.12 to -0.05; *P <*.001). Elevated internalizing symptoms at age 11 were associated with increased externalizing symptoms at age 14 (β = 0.04; *SE =* 0.02; 95% CI, 0.003–0.08; *P* =.046). The same path between ages 14–17 was not significant (Fig. 2).

Elevated externalizing symptoms at age 11 were associated with increased monthly alcohol use (β = 0.06; *SE =* 0.02; 95% CI, 0.02–0.10; *P* =.004) and internalizing symptoms (β = 0.12; *SE =* 0.02; 95% CI, 0.07–0.16; *P <*.001) at age 14. The same paths between ages 14–17 were not significant (Fig. 2).

The Cross-Lagged and Autoregressive Paths Between Alcohol Use and Internalizing/Externalizing Symptoms. This figure shows the paths (*p <.05, **p <.001), between monthly alcohol use frequency and internalizing/externalizing symptoms (presented in black) and between internalizing and externalizing symptoms (presented in grey). Solid lines represent significant paths. Dashed lines represent non-significant paths. Standardized estimates with 95% confidence intervals are presented for significant paths only.

Elevated alcohol use frequency at age 11 predicted increased internalizing symptoms at age 14 (β = 0.06; *SE =* 0.02; 95% CI, 0.01–0.11; *P =*.01), and increased monthly alcohol use at age 14 predicted increased externalizing symptoms at age 17 (β = 0.11; *SE =* 0.02; 95% CI, 0.08–0.15; *P <*.001). Monthly alcohol use at age 11 was not significantly associated with any changes in externalizing symptoms at age 14, nor was alcohol use at age 14 significantly associated with changes in internalizing symptoms at age 17 (Fig. 2).

Within-person contemporaneous correlations and associations between random intercepts, reflecting between-person trait-like differences, are reported in Table 2. The trivariate RI-CLPM showed positive significant autoregressive paths for internalizing and externalizing symptoms across ages 11 to 17, with diminishing carry-over effects over time. Interestingly, the monthly alcohol use autoregressive path was significant from ages 14 to age 17, but not between ages 11 and, indicating that stability in monthly alcohol use occurs in late, but not in early adolescence (Table 2).

**Table 2:** The within-person contemporaneous correlations and between-person associations between Random intercepts

	*r*	SE	95% CI	
			Lower	Upper
**Within-person contemporaneous correlations**				
* Age 11*				
Monthly alcohol ue ~ internalizing symptoms	0.11*	0.04	0.04	0.18
Monthly alcohol use ~ externalizing symptoms	0.06*	0.03	0.01	0.12
Internalizing symptoms ~ externalizing symptoms	0.38**	0.03	0.32	0.43
* Age 14*				
Monthly alcohol use ~ internalizing symptoms	0.01	0.02	-0.03	0.05
Monthly alcohol use ~ externalizing symptoms	0.09**	0.02	0.05	0.13
Internalizing symptoms ~ externalizing symptoms	0.30**	0.02	0.26	0.33
* Age 17*				
Monthly alcohol use ~ internalizing symptoms	-0.03	0.02	-0.06	0.001
Monthly alcohol use ~ externalizing symptoms	0.13**	0.02	0.10	0.16
Internalizing symptoms ~ externalizing symptoms	0.36**	0.02	0.33	0.40
**Between-person associations**				
Monthly alcohol use ~ internalizing symptoms	-0.28*	0.09	-0.45	-0.10
Monthly slcohol use ~ externalizing symptoms	-0.01	0.07	-0.15	0.13
Internalizing symptoms ~ externalizing symptoms	0.24	0.14	-0.03	0.51

*p <.05. **p <.001

### Influence of perinatal, parent and household-level CRIs

Table 3 and Supplementary Figures S2-S8 present all results of the conditional RI-CLPM model.

Perinatal CRI and early childhood adverse parenting showed no significant association with monthly alcohol use at any timepoint but did predict increased externalizing symptoms across all ages (Table 3, Supplementary Figures S2 and S3). Additionally, perinatal CRI predicted increased internalizing symptoms at age 17, whilst early childhood adverse parenting predicted increased internalizing symptoms at ages 11 and 14. Longitudinal parent-level risk occurrence was associated with elevated monthly alcohol use at ages 14 and 17, and increased internalizing and externalizing symptoms across all ages (Table 3, Supplementary Figure S4). Persistent household socioeconomic deprivation was associated with reduced monthly alcohol use at ages 14 and 17 (Table 3, Supplementary Figure S5). It showed a positive association with internalizing symptoms at ages 11 and 14, and with externalizing symptoms across all ages.

### Influence of sex and adolescent alcohol expectancies

Boys reported significantly more frequent monthly alcohol use than girls at age 11 (Table 3, Supplementary Figure S6). They also reported lower levels of internalizing, and higher levels of externalizing symptoms, across all ages. Positive alcohol expectancies were associated with elevated monthly alcohol use across all ages (Table 3, Supplementary Figure S7). Negative alcohol expectancies were associated with reduced monthly alcohol use at age 11 (Table 3, Supplementary Figure S8).

**Table 3:** The direct effects of sex, CRIs and alcohol expectances on monthly alcohol use, internalizing and externalizing symptoms

	Monthly alcohol use					Internalizing symptoms					Externalizing symptoms			
	β	SE	95% CI			β	SE	95% CI			β	SE	95% CI	
			Lower	Upper				Lower	Upper				Lower	Upper
	**Age 11**													
Male	0.04*	0.01	0.01	0.06		-0.07**	0.02	-0.10	-0.04		0.13**	0.02	-0.10	0.16
Perinatal CRI	0.01	0.02	-0.02	0.04		0.02	0.02	-0.01	0.05		0.05*	0.02	0.02	0.08
Longitudinal parent-pevel risk occurrence	0.02	0.02	-0.02	0.05		0.11**	0.02	0.08	0.14		0.15**	0.02	0.12	0.18
Persistent household SED	0.01	0.02	-0.02	0.04		0.08**	0.02	0.05	0.12		0.12**	0.01	0.10	0.15
Early childhood adverse parenting	-0.02	0.02	-0.05	0.01		0.14**	0.02	0.10	0.17		0.25**	0.02	0.22	0.28
Positive alcohol expectancies	0.11**	0.02	0.08	0.15		0.04*	0.02	0.01	0.07		0.02	0.02	-0.01	0.05
Negative alcohol expectancies	-0.06**	0.01	-0.09	-0.03		-0.01	0.01	-0.04	0.01		-0.05**	0.01	-0.08	-0.02
	**Age 14**													
Male	-0.02	0.02	-0.05	0.010		-0.16**	0.02	-0.19	-0.13		0.12**	0.02	0.09	0.15
Perinatal CRI	0.01	0.02	-0.02	0.04		0.02	0.02	-0.01	0.05		0.06**	0.01	0.03	0.09
Longitudinal parent-level risk occurrence	0.09**	0.02	0.05	0.12		0.11**	0.02	0.08	0.14		0.14**	0.01	0.12	0.17
Persistent household SED	-0.05**	0.01	-0.08	-0.03		0.10**	0.02	0.07	0.13		0.13**	0.01	0.11	0.16
Early childhood adverse parenting	0.01	0.02	-0.03	0.04		0.11**	0.02	0.08	0.14		0.20**	0.02	0.17	0.23
Positive alcohol expectancies	0.09**	0.01	0.07	0.12		-0.002	0.02	-0.03	0.03		-0.002	0.02	-0.03	0.03
Negative alcohol expectancies	-0.02	0.01	-0.05	0.01		-0.03	0.01	-0.06	0.00		-0.04*	0.01	-0.07	-0.02
	**Age 17**													
Male	0.02	0.01	-0.01	0.04		-0.34**	0.01	-0.37	-0.31		0.09**	0.02	0.06	0.13
Perinatal CRI	0.02	0.02	-0.01	0.05		0.03*	0.01	0.00	0.06		0.06**	0.02	0.03	0.09
Longitudinal parent-level risk occurrence	0.06**	0.02	0.03	0.10		0.06**	0.02	0.03	0.09		0.10**	0.01	0.07	0.13
Persistent household SED	-0.18**	0.01	-0.20	-0.15		0.02	0.01	0.00	0.05		0.05**	0.01	0.02	0.07
Early childhood adverse parenting	-0.03	0.02	-0.06	0.00		0.01	0.01	-0.02	0.03		0.09**	0.01	0.06	0.11
Positive alcohol expectancies	0.09**	0.02	0.05	0.12		0.03*	0.01	0.00	0.06		0.07**	0.02	0.03	0.10
Negative alcohol expectancies	0.01	0.02	-0.03	0.04		0.01	0.01	-0.02	0.04		0.01	0.02	-0.02	0.04

*p <.05. **p <.001

## Discussion

To our knowledge, the current study is the first to uncover a reciprocal relationship between adolescent mental health difficulties and frequent alcohol consumption between 11 and 17 years. We found a significant reciprocal association between more externalizing symptoms and more frequent monthly alcohol use from the ages of 11 to 17, providing novel evidence of links between alcohol use and externalizing disorders already during adolescence.

More specifically, we found that increased externalizing symptoms in early adolescence (age 11) predicted increased alcohol use at ages 14–17, which in turn predicted elevated externalizing symptoms at age 17. These results are consistent with, and expand upon previous findings showing that externalizing symptoms represent a risk factor for increased alcohol use in adulthood [9]. Hence, the findings lend support to the externalizing pathway to comorbid AUDs and externalizing disorders, which suggests that the behavioural disinhibition often associated with externalizing symptoms increases adolescents’ propensity for engaging in deviant behaviour, like underage drinking [31]. Furthermore, while little research has explored the possible underlying mechanisms of alcohol as a risk factor for externalizing symptoms, available studies have shown that adolescents carrying a polymorphism of the aldehyde dehydrogenase 2 (ALHD2) gene commonly associated with reduced alcohol consumption [32], were also less likely to report aggressive behaviour or attentional deficits during adolescence [33]. Therefore, our findings lend further support to theoretical models positing that the potentiated neurotoxic effects of alcohol on the developing adolescent brain might elicit neuroadaptations in regions implicated in the pathogenesis of mental health difficulties [34]. Overall, our results suggest that externalizing symptomatology and alcohol consumption serve to maintain and/or exacerbate one another throughout adolescence.

Our results also showed a reciprocal relationship between internalizing symptoms and alcohol use from the ages of 11 to 17. While increased monthly alcohol use during early adolescence (11-14yrs) predicted more internalizing symptoms at age 14, more internalizing symptoms predicted reduced monthly alcohol consumption across adolescence. This is in line with previous research linking adolescent alcohol consumption, even at subclinical levels, to an increased risk for developing depressive symptoms in adulthood [8]. Our results expand upon previous research in the field by showing that the link between alcohol consumption and internalizing symptomatology already exists in adolescence. Conversely, the finding that more internalizing symptoms consistently predicted a reduced likelihood of engaging in frequent alcohol consumption contradicted our expectations. Previous studies show mixed results on the relationship between internalizing disorders and alcohol consumption [12, 14], and this may be due to the observed relationship between higher internalizing and higher externalizing symptoms [29]. It is possible that when externalizing symptoms are controlled for, internalizing symptoms are related to reduced alcohol consumption. In support of this, Nurnberger and colleagues [35], found that adolescent externalizing disorders predicted an earlier onset of AUD in early adulthood. However, regarding internalizing disorders, this association was only significant in the presence of a co-occurring externalizing disorder. As adolescent drinking often occurs in social contexts with peers [36], it is possible that the elevated levels of social withdrawal associated with internalizing symptoms [37], may inadvertently reduce social opportunities for frequent alcohol consumption. It is plausible that the motivation to drink to cope with negative emotionality, hypothesized to underlie the increased risk of AUD resulting from internalizing symptoms, only develops in adulthood, rather than during the initiation/escalation of alcohol use during adolescence [34]. Thus, disparities in previous research findings may in part be due to the influence of developmental timing on the temporal relationship between internalizing symptoms and alcohol use. In support of this, research suggests that the protective influence of internalizing symptoms diminishes with age [38].

In terms of the risk factors we controlled for, we found that exposure to more parental risk factors, such as parental alcohol or drug consumption, domestic violence, or poor parental mental health before 11 years was significantly associated with higher levels of adolescent alcohol use and mental health difficulties, consistent with existing literature [39, 40]. Interestingly, adolescents from higher socioeconomic backgrounds were more likely to report frequent alcohol use, consistent with research conducted in this age group in other British samples [41].

Also, and in line with previous findings (see Smit et al. for a review) [27], positive alcohol expectancies, such as the belief in enhanced confidence and sociability, during early adolescence, predicted increased alcohol use across all ages. In contrast, negative expectancies, such as the belief that drinking hinders schoolwork, only predicted reduced alcohol use at age 11. Overall, the findings underscore the crucial role of positive alcohol expectancies as a modifiable risk factor for the initiation/escalation of underage drinking throughout adolescence. Additionally, in accordance with the literature, boys reported more frequent monthly alcohol use at age 11 [42]. Boys also reported higher levels of externalizing, and lower levels of internalizing symptoms across all ages [43], compared to girls.

### Limitations

The current study relied on a single-item measure of alcohol use frequency. This has been found to be effective method of screening for problematic adolescent alcohol consumption [44]. However, while previous research shows that frequent adolescent alcohol consumption reflects a risk factor for subsequent AUDs and psychiatric disorders in adulthood [6–8], the relationship between adolescent alcohol use and mental health difficulties may differ depending on the dimension of adolescent drinking behaviour that was measured [45]. Thus, future research should explore other dimensions, such as the frequency of heavy episodic drinking, for a more nuanced understanding of the temporal relationship between various facets of adolescent drinking behaviour and mental health difficulties.

### Implications

The current findings emphasize the significance of adolescent alcohol use as a risk factor for subsequent mental health difficulties, indicating that early screening in adolescence followed by preventative interventions against underage drinking also may ameliorate the risk of future mental health difficulties. Screening for externalizing disorders in childhood and early adolescence may enable the early identification of adolescents at a higher risk of engaging in frequent underage drinking. Targeted interventions to address externalizing symptomatology prior to alcohol initiation may also diminish the risk of underage drinking. Initial evidence in the field of attention-deficit/hyperactivity disorder (ADHD) research may inform such strategies. Indeed, stimulant medications for children with ADHD have been found to at reduce both externalizing symptomatology and the risk of future substance use [46].

Additionally, evidence from this study may inform future strategies aimed at preventing the development of comorbid AUDs and externalizing disorders. The interconnected nature of externalizing symptoms and alcohol use during adolescence point to the need for a unified approach. Alcohol screening and brief intervention (SBI) has been shown as a cost-effective intervention with demonstrated efficacy for reducing adolescent alcohol consumption [47]. Therefore, incorporating SBI into adolescent mental health treatment settings could facilitate the early identification and referral of adolescents with high levels of externalizing symptoms and problematic alcohol consumption to substance abuse treatment services. This approach may help to reduce the risk of future comorbid AUDs and externalizing disorders in early adulthood.

## Conclusions

Our findings revealed that frequent adolescent alcohol use posed a risk for both higher externalizing and internalizing symptoms, while higher internalizing symptoms were associated with less frequent alcohol use across adolescence. Additionally, the study extends the existing evidence implicating externalizing symptoms as a risk factor for frequent alcohol consumption in adolescence by uncovering the existence of a reciprocal relationship between externalizing symptoms and alcohol use frequency. Overall, our findings provide a strong rationale for additional research assessing the implementation of routine screening, followed by an appropriate evidence-based intervention to reduce alcohol consumption, for adolescents presenting to mental health services as an effective way to prevent AUDs and psychiatric conditions in adulthood.

## Electronic supplementary material

Below is the link to the electronic supplementary material.

Supplementary Material 1

## Data Availability

The Millennium Cohort Study dataset can be accessed via the UK Data Service. The R script and output for the RI-CLPM and the SPPS syntax needed to compute the CRI variables are available on Pure.
