# Supplementary material for: Reciprocal relationships between adolescent mental health difficulties and alcohol consumption
Source: Eur Child Adolesc Psychiatry. 2025 Jan 18;34(8):2347–57. doi: 10.1007/s00787-025-02644-6 (PMC12397185; doi:10.1007/s00787-025-02644-6)
Supplement: Supplementary file 1 — Supplementary Material 1 [file 787_2025_2644_MOESM1_ESM.docx]

**Article Title:**

Reciprocal Relationships between Adolescent Mental Health Difficulties and Alcohol Consumption

**Journal Name:**

European Child & Adolescent Psychiatry

**Author Names:**

Janet Kiri, MSc, James Hall, PhD, Samuele Cortese, MD, PhD, Valerie Brandt, PhD

**Corresponding Author:**

Janet Kiri, School of Psychology, Centre for Innovation in Mental Health, University of Southampton, Southampton, UK

**Email for Corresponding Author:** jk3g18@soton.ac.uk

**Supplementary Material**

**Table of Contents**

[**Supplement 1. Description of the Random-Intercept Cross-Lagged Panel Model 3**](#_Toc183858548)

[**Table S1. Description of Psychometric Inventories 4**](#_Toc183858549)

[**Table S2. Variables Included in Cumulative Risk Indices 6**](#_Toc183858550)

[**Table S3. Correlation Matrix for Monthly Alcohol Use, Internalizing and Externalizing Symptoms 9**](#_Toc183858551)

[**Figure S1. A Flow Chart of the Inclusion/Exclusion Criteria. 10**](#_Toc183858552)

[**Figure S2. The Significant Effects of Perinatal Risk 11**](#_Toc183858553)

[**Figure S3. The Significant Effects of Early Childhood Adverse Parenting 12**](#_Toc183858554)

[**Figure S4. The Significant Effects of Longitudinal Parent-Level Risk Occurrence 13**](#_Toc183858555)

[**Figure S5. The Significant Effects of Persistent Household Socioeconomic Deprivation 14**](#_Toc183858556)

[**Figure S6. The Significant Effects of Sex 15**](#_Toc183858557)

[**Figure S7. The Significant Effects of the Positive Alcohol Expectancies 16**](#_Toc183858558)

[**Figure S8. The Significant Effects of the Negative Alcohol Expectancies 17**](#_Toc183858559)

[**References 18**](#_Toc183858560)

**Supplement 1. Description of the Random-Intercept Cross-Lagged Panel Model**

A random-intercept cross-lagged panel model (RI-CLPM) [1, 2] was employed to explore the dynamic relationship between monthly alcohol use, internalizing and externalizing symptoms across three timepoints (11yrs, 14yrs and 17yrs), controlling for sex and various salient risk factors. Previous research utilizing the traditional CLPM may have produced spurious, and/or have failed to detect, significant cross-lagged effects as, unlike with the RI-CLPM, the relationships are confounded by between-person trait-like differences [1, 2]. RI-CLPM demonstrates significant advantages over the traditional CLPM through including random intercepts which enables the delineation of between-person from within-person variances. Thus, the cross-lagged component in RI-CLPM may provide more accurate insight into the longitudinal relationship between alcohol use and internalizing/externalizing symptoms.

Cross-lagged effects in a RI-CLPM describe how deviations from an individual’s baseline level of one construct predict subsequent deviations in another construct. Therefore, the cross-lagged paths reflect within-person associations that represent how an increase or decrease in one variable (e.g. internalizing/externalizing symptomatology) is associated with a subsequent increase or decrease in the other variable (e.g. the frequency of alcohol consumption), within the same individual. Similarly, autoregressive effects describe the persistence of changes within a variable over time relative to an individual’s baseline. On the other hand, the random intercepts capture stable individual differences in internalizing and externalizing symptomatology and the frequency of alcohol consumption across time, and the covariances between these random intercepts reflect the time-invariant relationship between individuals’ trait-like levels of these constructs [1, 2].

A trivariate RI-CLPM was conducted to adjust for the high co-occurrence between internalizing and externalizing symptoms [3], facilitating an understanding of their potential reciprocal relationships with alcohol use frequency over time. Detailed descriptions of the RI-CLPM compared to other longitudinal cross-lagged models often used in the literature to investigate prospective effects have been provided elsewhere [4, 5].

**Table S1. Description of Psychometric Inventories**

| **Measure** | **Description** |
| --- | --- |
| Strengths and Difficulties Questionnaire | The 25-item Strengths and Difficulties Questionnaire (SDQ) was used as a measure of childhood emotional and behavioural problems at T5-T7. The SDQ comprises five subscales, each consisting of five items; emotional problems (*e.g. “Many fears, easily scared”*), prosocial behaviour (*e.g.* *“Shares readily with others”*), hyperactivity/inattention (*e.g. “Restless, overactive, can not stay still for long”*), conduct problems (*e.g.* *“Can be spiteful to others”),* and peer problems (*e.g.* *“Picked on or bullied by other children”*) [6]. Parents and cohort-members indicated their level of agreement to 25 statements about the adolescent on the scale (0) “Not true”, (1) “Somewhat true”, and (2) “Certainly true”. The parent- report of the SDQ was used at ages 11 and 14 as previous research has demonstrated it is a valid instrument for comparing mental health difficulties in young people between these ages [7, 8]. The adolescent self-report of the SDQ was used at age 17 due to a lack of measurement invariance in the parent-reported SDQ, as well as the adolescents’ increasing independence and greater ability to self-report their mental health symptoms at this age [7, 8]. The parent-reported emotional problems (α = 0.76), conduct problems (α = 0.59), and hyperactivity/inattention (α = 0.76) subscales, as well as the adolescent’s self-reported emotional problems (alpha = 0.74), conduct problems (α = 0.56), and hyperactivity/inattention (α = 0.73) demonstrated acceptable internal reliability in previous research conducted in the MCS [9]. For our purposes, internalizing symptoms were measured using the emotional problems subscale (range; 0-10), externalizing symptoms were measured with the hyperactivity/inattention and conduct problems subscales (range: 0-20). Higher scores indicate a greater number of symptoms. |
| Rutter Malaise Inventory | Parental psychological distress was measured using the 9-item Rutter Malaise Inventory at T1 [10]. Parents indicated either (1) "Yes" or (0) "No" to 9 questions about emotional and somatic symptoms of depression and anxiety (*e.g. “Do you often feel miserable or depressed?”*). All items were summed to create a total score ranging from 0-9, with higher scores indicating a higher degree of psychological distress. The 9-item version of the Rutter Malaise Inventory has demonstrated acceptable internal consistency (α = 0.70) in previous research [11]. |
| Kessler 6 | Parental psychological distress was measured using the Kessler 6 (K6) scale at T2-T5 [12]. Parents indicated their level of agreement with 6 statements about their emotional state (*e.g. “During the last 30 days, about how often did you feel so depressed that nothing could cheer you up?”*), on a 5-point scale (0) "None of the time", (1) “A little of the time”, (2) “Some of the time”, (3) “Most of the time” and (4) "All of the time". A total score was created ranging from 0-24 with higher scores indicating a higher degree of psychological distress. The K6 has demonstrated acceptable internal consistency (α = 0.89) in previous research [12]. |
| Pianta Child-Parent Relationship Scale | The Child-Parent Relationship Scale is a 14 item scale that measures the main caregiver’s self-reported feelings and beliefs about the relationship with their child at T2 (3 years old). [13] Main caregiver’s indicated their level of agreement with 14 statements on a 5-point scale, (0) "Definitely does not apply", (1) “Not really”, (2) “Neutral”, (3) “Applies Sometimes”, and (4) “Definitely applies”. The scale comprises two subscales, each comprised of 7-items; perceived conflict (*e.g. “uncomfortable with physical affection”*) and perceived warmth (*e.g. “will seek comfort from me if upset”*). The items for each subscale were summed to create a total score ranging from 0-42, with higher scores indicating a higher degree of maternal warmth or conflict in the parent-child relationship. The warmth (α = 0.72) and conflict (α = 0.78) subscales have demonstrated acceptable internal consistency in this cohort.[11] |
| Straus Conflict Tactics Scale | Harsh parenting was assessed at T2 using the Straus Conflict Tactics scale which comprises a 6-item measure of parental discipline practices [14]. Parents were asked how often they used active discipline tactics (*e.g. “smacking”*) and withdrawal tactics (*e.g. “ignoring the child”*) when the child was naughty. Responses were provided on the scale (0) "Never", (1) “Rarely”, (2) “Once a month”, (3) “Once a week or more”, and (4) "Daily". A dichotomous variable was created with responses (2) "Once a month", (3) "Once a week or more" and (4) "Daily" coded as (1) "Yes” and responses of (0) "Never" and (1) "Rarely" coded as (0) "No". A total score was created with higher scores indicating a higher use of harsh parenting practices. |
| Home Learning Environment | The Home Learning Environment Index was administered at T2 and comprised 6 items related to the frequency the parent engaged the child in a range of learning activities (*e.g. "going to the library"*) [15]. Responses to the item “going to the library” were provided on the scale (0) “Not at all”, (1) “On Special occasions”, (3) “Once a Month”, (5) “Once a fortnight”, (7) “Once a week”. Responses to the item “read to the child” were provided on the scale (0) “Not at all”, (1) “Less often than once or twice a month”, (3) “Once or twice a month”, (5) “Once or twice a week”, (6) “Several times a week”, (7) “Every day”. For the remaining four items (“painting and drawing”, “being taught letters”, “being taught numbers”, “songs/poems/rhymes”) responses were provided on the scale (0) "Not at all", (1) “Occasionally or less than once a week”, (2) “1-2 days per week”, (3) “3 times a week”, (4) “4 times a week”, (5) “5 times a week”, (6) “6 times a week”, (7) "Everyday". A total score was created ranging from 0-42, with higher scores indicating a higher quality home learning environment. |
| Alcohol Expectancies | Positive (*e.g. “Drinking alcohol makes people happier with themselves”*) and negative (e.g. *“Drinking alcohol gets in the way of school work”*) alcohol expectancies at age 11 were assessed via self-report with four and three items respectively, using a scale that was adapted for an adolescent British population (from Guo and colleagues) [16]. Responses were provided on the scale (0) "strongly disagree", (1) “Disagree”, (2) “Agree”, (3) "Strongly agree". The score for each subscale was averaged to indicate the adolescents' beliefs about the effects of drinking [17], with higher scores reflecting higher levels of positive or negative alcohol expectancies respectively. |

**Table S2. Variables Included in Cumulative Risk Indices**

|  | **No. (%) with data** | **Mean (SD)** | **Cut-off for high-risk** | **No. (%) high-risk** | |
| --- | --- | --- | --- | --- | --- |
| **Perinatal Cumulative Risk Index^a^** |  |  |  |  | |
| Birth weight [18, 19] | 10253 (96.3%) | 3.35 (0.6) | < 2.5kg | 752 (7.1%) | |
| Gestation [20, 21] | 10156 (95.4%) | 275.75 (14.1) | <= 252 days gestation | 648 (6.1%) | |
| Pregnancy alcohol consumption [22, 23] | 10253 (96.3%) |  | Any alcohol consumption | 3139 (29.5%) | |
| Everyday | 36 (0.4%) |  |  |  | |
| 5-6 times per week | 25 (0.2%) |  |  |  | |
| 3-4 times per week | 129 (1.3%) |  |  |  | |
| 1-2 times per week | 756 (7.4%) |  |  |  | |
| 1-2 times per month | 752 (7.3%) |  |  |  | |
| Less than once a month | 1441 (14.1%) |  |  |  | |
| Never | 7114 (69.4%) |  |  |  | |
| Pregnancy smoking status [24, 25] | 9576 (89.9%) |  | More than one cigarette smoked | 2621 (24.6%) | |
| Maternal BMI pre-pregnancy [26, 27] | 9408 (88.4%) | 23.75 (4.45) | BMI >= 30kg/m2 - Obesity class I, II or III | 845 (7.9%) | |
| Smoking in same room as child [28, 29] | 10266 (96.4%) |  | Any smoking in the same room as cohort member from birth to 9 months old | 1231 (11.6%) | |
|  |  |  |  |  | |
| Perinatal Cumulative Risk Index | 10254 (96.3%) |  | Percentage encountering: |  | |
|  |  |  | 4+ risks | 98 (1%) | |
|  |  |  | 3 risks | 486 (4.7%) | |
|  |  |  | 2 risks | 1705 (16.6%) | |
|  |  |  | 1 risk | 3966 (38.7%) | |
|  |  |  | No risk | 3999 (39%) | |
| **Early Childhood Adverse Parenting^b^** |  |  |  |  | |
| Harsh Discipline Practices [30, 31] | 7731 (72.6%) | 3.13 (1.58) | ≥ 4 Total score on Straus Conflict Tactics Scale[14] | 1645 (15.5%) | |
| Maternal Warmth [32, 33] | 8869 (83.3%) | 26.51 (2.36) | ≤ 24 Total score on Pianta Parent-Child Relationship (warmth subscale)[13] | 1247 (11.7%) | |
|  | **No. (%) with data** | **Mean (SD)** | **Cut-off for high-risk** | **No. (%) high-risk** | |
| Maternal Conflict [34, 35] | 8998 (84.5%) | 8.94 (5.7) | ≥ 12 Total score on Pianta Parent-Child Relationship (conflict subscale)[13] | 2800 (26.3%) | |
| Home Learning Environment [36, 37] | 9725 (91.3%) | 26.07 (7.36) | Bottom Quartile[15] | 1888 (17.7%) | |
| Early Childhood Adverse Parenting Cumulative Risk Index | 8937 (83.9%) |  | Percentage encountering: |  | |
|  |  |  | 3+ risks | 405 (4.5%) | |
|  |  |  | 2 risks | 1445 (16.2%) | |
|  |  |  | 1 risk | 3063 (34.3%) | |
|  |  |  | No risk | 4024 (45%) | |
| **Longitudinal Parent-Level Risk Occurrence^c^** | | | | |  |
| Frequent Parental Alcohol Consumption [38, 39] | 10646 (100%) |  | ≥ 5-6 times per Week | 5007 (47%) | |
| Parental Drug Use [40, 41] | 10111 (95%) |  | Any drug use | 1225 (11.5%) | |
| Parental Smoking [42, 43] | 10646 (100%) |  | Parent smokes any tobacco | 5475 (51.4%) | |
| Recipient of Domestic Violence [44, 45] | 9868 (92.7%) |  | Report of domestic violence | 2111 (19.8%) | |
| Poor Mental Health [46, 47] | 10600 (99.6%) |  | ≥ 13 total score on the Kessler (6 item) Scale,[12] and/or ≥ 4 total score on the Rutter Malaise Inventory[10] | 2081 (19.5%) | |
| Low Maternal/PCG (Highest) Occupational Status [48, 49] | 10399 (97.7%) |  | Semi-Skilled or Lower | 5053 (47.5%) | |
| Low Maternal/PCG (Highest) Education Level [50, 51] | 10046 (94.4%) |  | No High-School Qualification | 1256 (11.8%) | |
|  |  |  |  |  | |
| Longitudinal Parent-Level Risk Occurrence | 10608 (99.6%) |  | Percentage encountering: |  | |
|  |  |  | 6+ risk | 95 (0.9%) | |
|  |  |  | 5 risks | 354 (3.3%) | |
|  |  |  | 4 risks | 1085 (10.2%) | |
|  |  |  | 3 risks | 2172 (20.5%) | |
|  |  |  | 2 risks | 3060 (28.8%) | |
|  |  |  | 1 risk | 2850 (26.9%) | |
|  |  |  | No risk | 992 (9.4%) | |
| **Persistent Household SED^d^** |  |  |  |  | |
| Low Household (Highest) Education Level [50, 51] | 10073 (94.6%) |  | No High-School Qualification | 1313 (12.3%) | |
| Low Household (Highest) Occupational Status [48, 49] | 10399 (97.7%) |  | Semi-Skilled or Lower | 1268 (11.9%) | |
| Low Household Income [52, 53] | 10646 (100%) |  | Below 60% poverty indicator | 1361 (12.8%) | |
| Persistent Household SED | 10588 (99.4%) |  | Percentage encountering: |  | |
|  |  |  | 2+ risks | 1046 (9.9%) | |
|  |  |  | 1 risk | 1596 (15.1%) | |
|  |  |  | No risk | 7946 (75%) | |

^a^The perinatal CRI assessed exposure to perinatal risk factors throughout pregnancy and up to when the cohort member was 9 months old.

^b^EC adverse parenting measures exposure to negative parenting styles during early childhood when the cohort member was 3 years old (T2).

^c^Longitudinal parent-level risk occurrence comprises a longitudinal measure that reflects exposure to parent-level risk factors up to T5 (11 years).

^d^Persistent household SED captured the continual exposure to socioeconomic deprivation at each available timepoint up to T5 (11 years).

**Table S3. Correlation Matrix for Monthly Alcohol Use, Internalizing and Externalizing Symptoms**

| **Variable** | **1.^a^** | **2.** | **3.** | **4.** | **5.** | **6.** | **7.** | **8.** | **9.** |
| --- | --- | --- | --- | --- | --- | --- | --- | --- | --- |
| 1. Monthly Alcohol Use (Age 11)^a^ | -- |  |  |  |  |  |  |  |  |
| 2. Monthly Alcohol Use (Age 14) | .11** | -- |  |  |  |  |  |  |  |
| 3. Monthly Alcohol Use (Age 17) | .09** | .31** | -- |  |  |  |  |  |  |
| 4. Internalizing Symptoms (Age 11) | -0.001 | -.04** | -.13** | -- |  |  |  |  |  |
| 5. Internalizing Symptoms (Age 14) | 0.01 | -.03** | -.13** | .56** | -- |  |  |  |  |
| 6. Internalizing Symptoms (Age 17) | -0.02 | 0.02 | 0.001 | .20** | .28** | -- |  |  |  |
| 7. Externalizing Symptoms (Age 11) | .06** | .04** | -.06** | .42** | .32** | .04** | -- |  |  |
| 8. Externalizing Symptoms (Age 14) | .05** | .08** | -.05** | .32** | .41** | .05** | .72** | -- |  |
| 9. Externalizing Symptoms (Age 17) | .04** | .12** | .15** | .12** | .12** | .32** | .30** | .36** | -- |

*** p < .001*

*^a^Spearman's rho correlations presented.*

# Figure S1. A Flow Chart of the Inclusion/Exclusion Criteria.

Completed at least one measure of SDQ or alcohol use at any timepoint between T5 and T7^a^

(N = 14,255)

Did not complete at least one measure of SDQ or alcohol use across all timepoints

(N = 5,228)

Analytical Sample

(N = 10,647)

Survey Weights Available for T7

(N = 10,653)

No Survey Weight Available for T7

(N = 3,602)^b^

Endorsed fake drug item at T7 (N = 6)

Enrolled in Study across all waves (N = 19,483)

The figure depicts the process of excluding participants from the overall sample of N = 19,483. Three exclusions were made in the current study to obtain the final analytical sample of N= 10,647.

^a^Participants were included at this stage if they provided at least one measure of the SDQ (or alcohol use) across any of the three timepoints (T5-T7) modelled in the RI-CLPM.

^b^See the Millennium Cohort Study (Sweep 7, Age 17) User Guide for further information regarding the computation of survey weights [54].

#
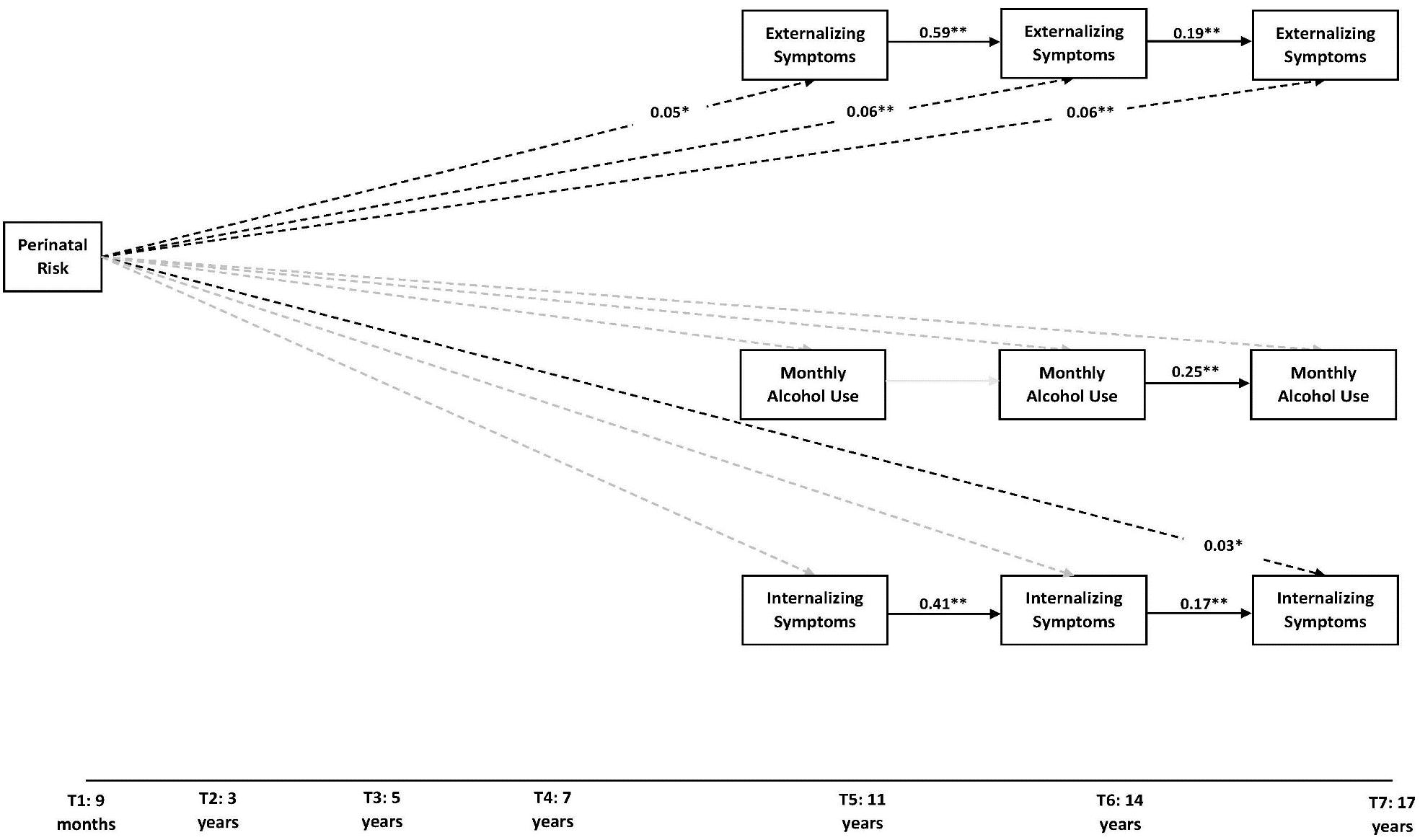
Figure S2. The Significant Effects of Perinatal Risk

T1: timepoint one (same pattern for subsequent timepoints).

The figure shows the significant autoregressive effects (black solid lines) from internalizing symptoms to the next wave of internalizing symptoms (T+1), from externalizing symptoms to the next wave of externalizing symptoms (T+1) and from monthly alcohol use to the next wave of monthly alcohol use (T+1). Significant effects from the perinatal CRI onto the internalizing and externalizing symptoms variables are included in black (dashed lines). Non-significant effects are presented in gray. All CRIs and covariates were adjusted for in the model simultaneously.

#
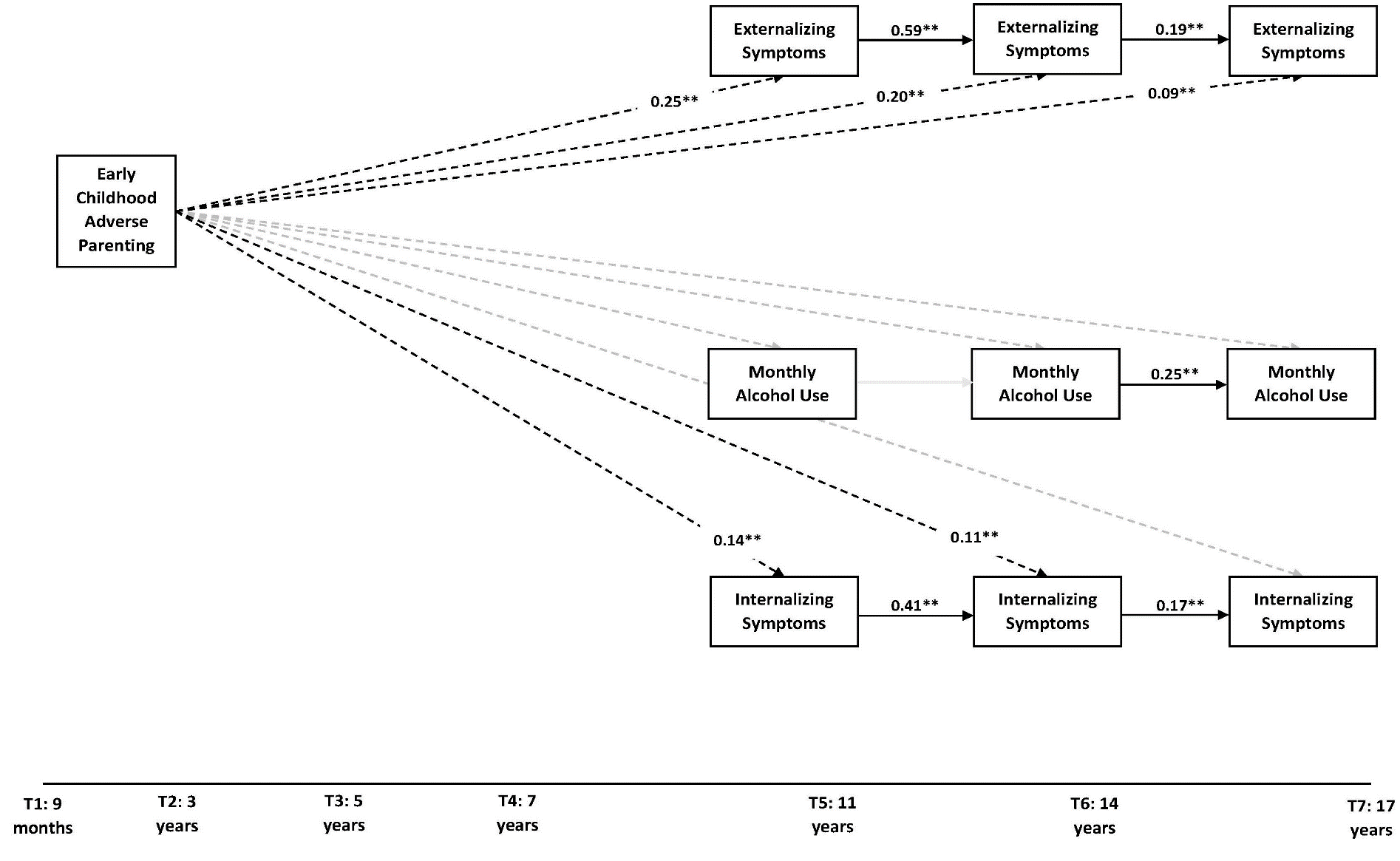
Figure S3. The Significant Effects of Early Childhood Adverse Parenting

T1: timepoint one (same pattern for subsequent timepoints).

The figure shows the significant autoregressive effects (black solid lines) from internalizing symptoms to the next wave of internalizing symptoms (T+1), from externalizing symptoms to the next wave of externalizing symptoms (T+1) and from monthly alcohol use to the next wave of monthly alcohol use (T+1). Significant effects from the early childhood adverse parenting CRI onto the internalizing and externalizing symptoms variables are included in black (dashed lines). Non-significant effects are presented in gray. All CRIs and covariates were adjusted for in the model simultaneously.

#
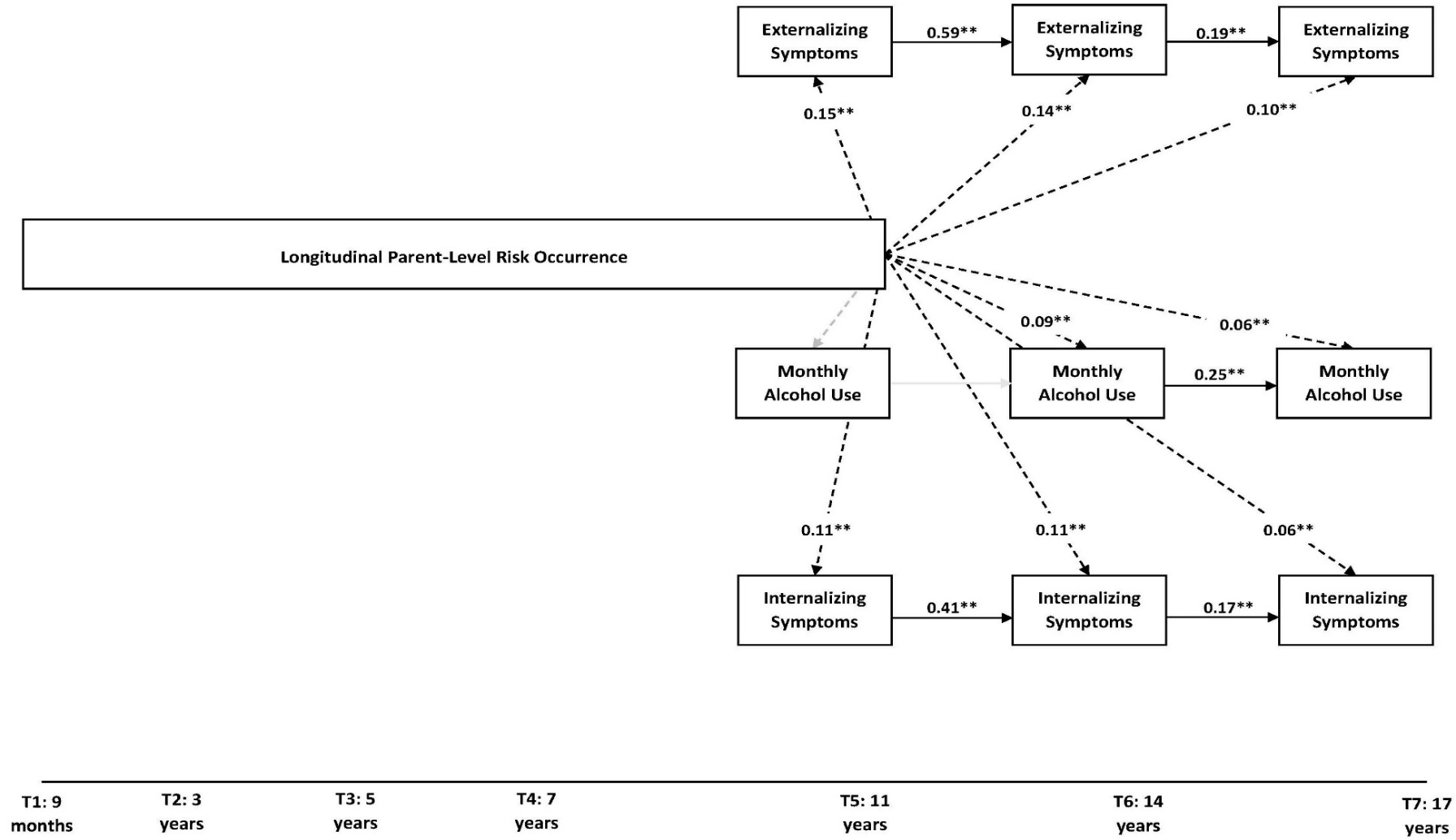
Figure S4. The Significant Effects of Longitudinal Parent-Level Risk Occurrence

T1: timepoint one (same pattern for subsequent timepoints).

The figure shows the significant autoregressive effects (black solid lines) from internalizing symptoms to the next wave of internalizing symptoms (T+1), from externalizing symptoms to the next wave of externalizing symptoms (T+1) and from monthly alcohol use to the next wave of monthly alcohol use (T+1). Significant effects from the longitudinal parent-level risk occurrence onto the monthly alcohol use, internalizing and externalizing symptoms variables are included in black (dashed lines). Non-significant effects are presented in gray. All CRIs and covariates were adjusted for in the model simultaneously.

# Figure S5. The Significant Effects of Persistent Household Socioeconomic Deprivation


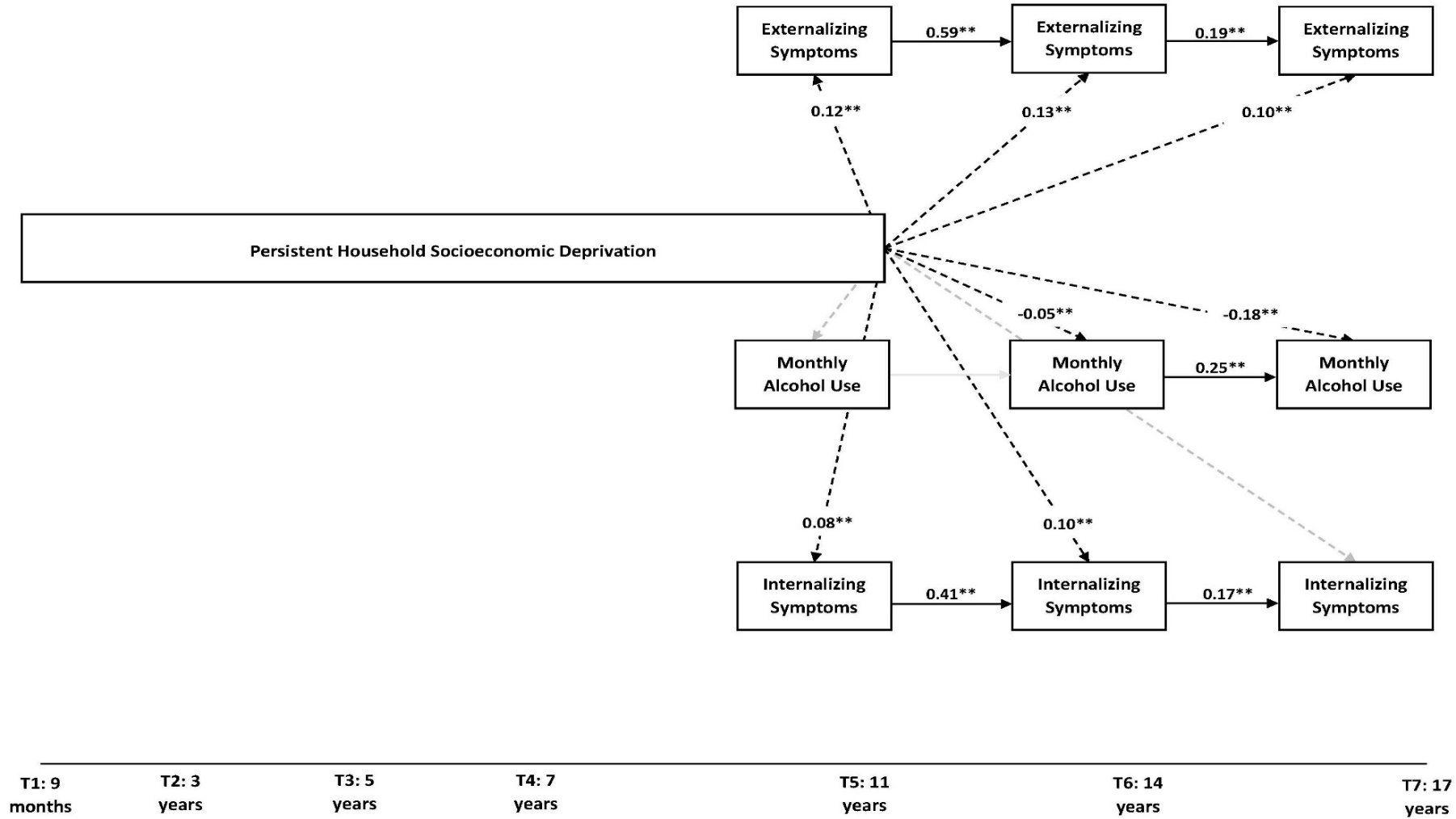


T1: timepoint one (same pattern for subsequent timepoints).

The figure shows the significant autoregressive effects (black solid lines) from internalizing symptoms to the next wave of internalizing symptoms (T+1), from externalizing symptoms to the next wave of externalizing symptoms (T+1) and from monthly alcohol use to the next wave of monthly alcohol use (T+1). Significant effects from the persistent socioeconomic deprivation onto the monthly alcohol use, internalizing and externalizing symptoms variables are included in black (dashed lines). Non-significant effects are presented in gray. All CRIs and covariates were adjusted for in the model simultaneously.

**
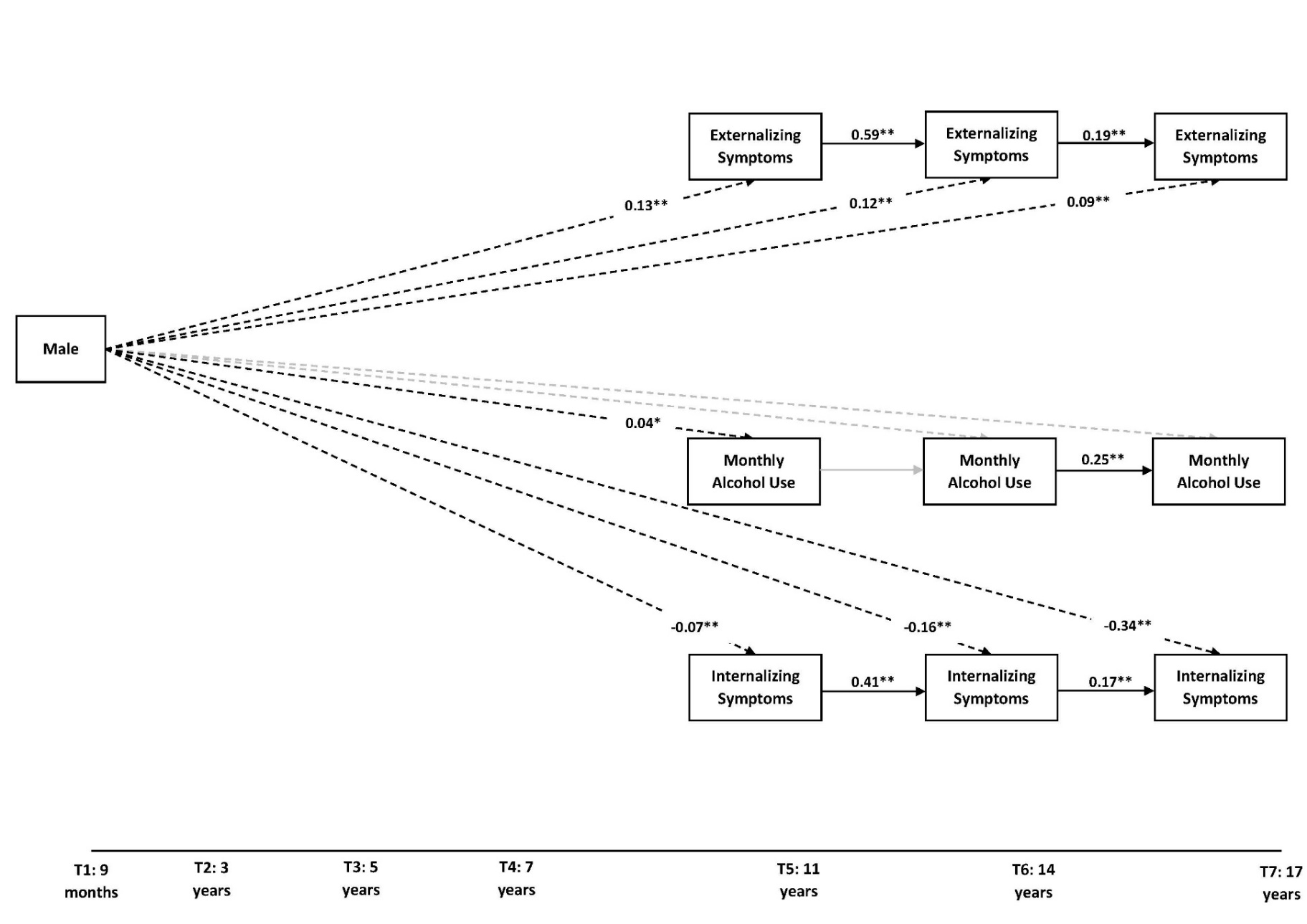
Figure S6. The Significant Effects of Sex**

T1: timepoint one (same pattern for subsequent timepoints).

The figure shows the significant autoregressive effects (black solid lines) from internalizing symptoms to the next wave of internalizing symptoms (T+1), from externalizing symptoms to the next wave of externalizing symptoms (T+1) and from monthly alcohol use to the next wave of monthly alcohol use (T+1). Significant effects of sex (male as reference category) onto the monthly alcohol use, internalizing and externalizing symptoms variables are included in black (dashed lines). Non-significant effects are presented in gray. All CRIs and covariates were adjusted for in the model simultaneously.

# Figure S7. The Significant Effects of the Positive Alcohol Expectancies


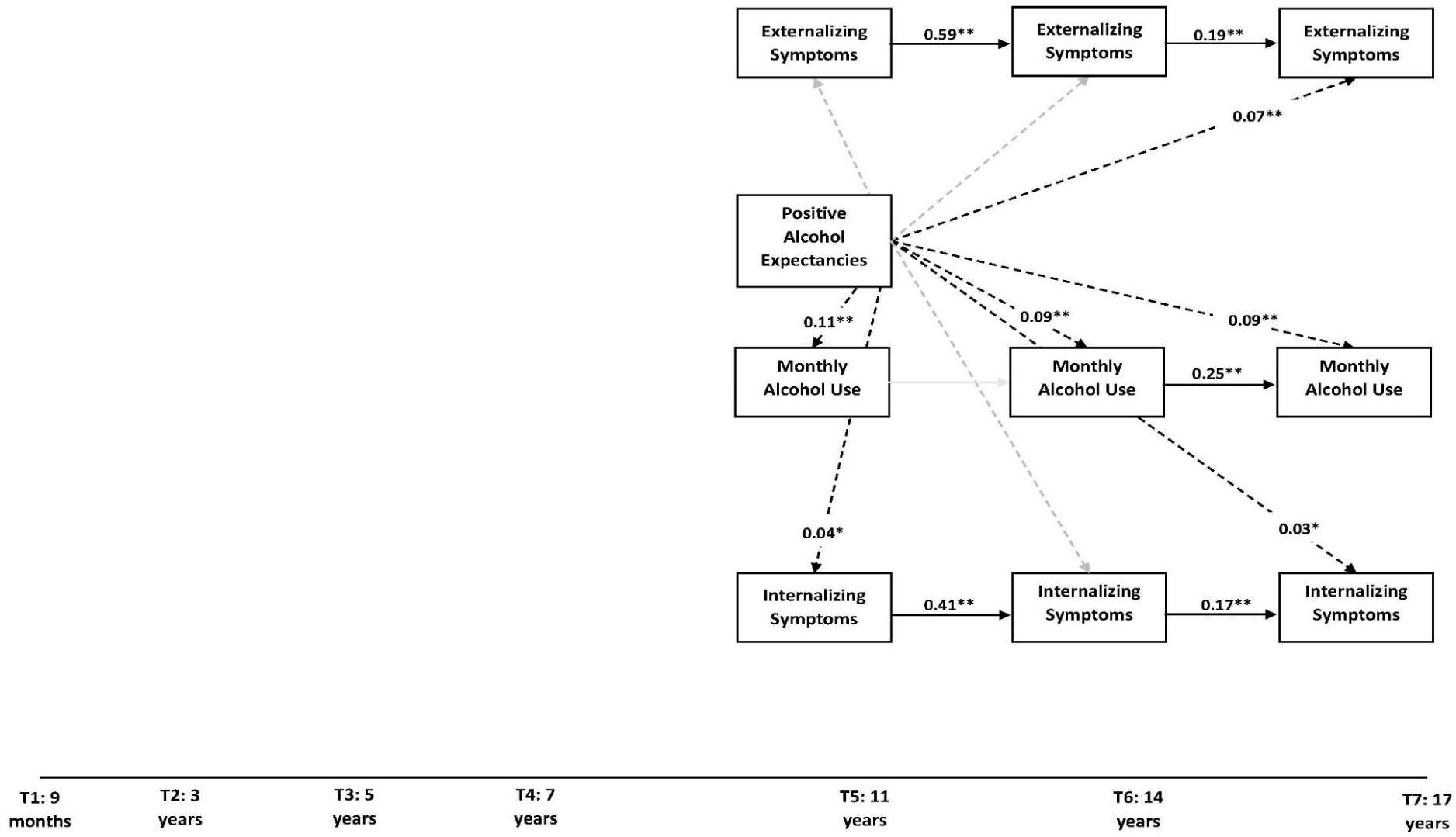


T1: timepoint one (same pattern for subsequent timepoints).

The figure shows the significant autoregressive effects (black solid lines) from internalizing symptoms to the next wave of internalizing symptoms (T+1), from externalizing symptoms to the next wave of externalizing symptoms (T+1) and from monthly alcohol use to the next wave of monthly alcohol use (T+1). Significant effects from positive alcohol expectancies onto the monthly alcohol use, internalizing and externalizing symptoms variables are included in black (dashed lines). Non-significant effects are presented in gray. All CRIs and covariates were adjusted for in the model simultaneously.

# Figure S8. The Significant Effects of the Negative Alcohol Expectancies


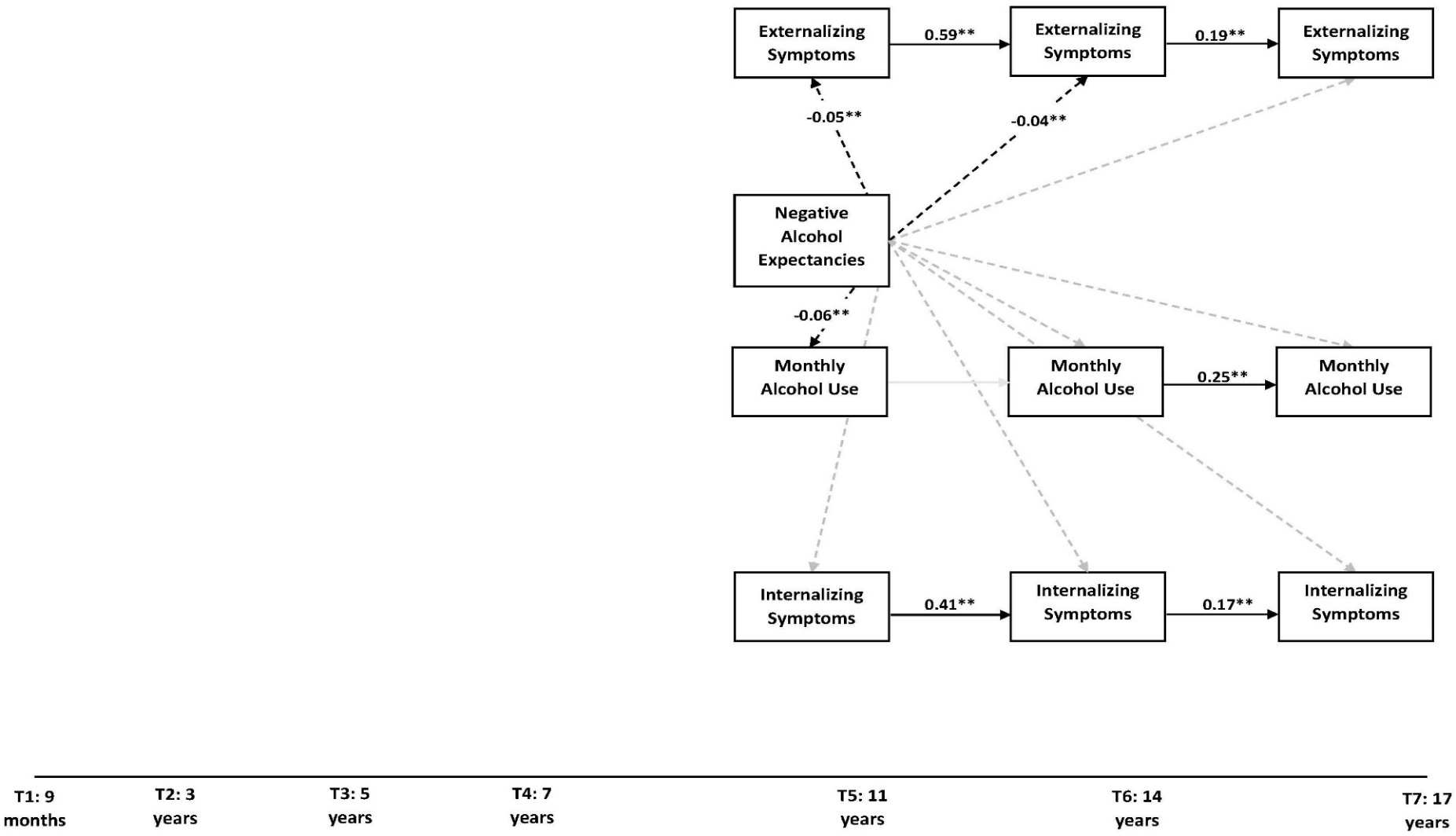


T1: timepoint one (same pattern for subsequent timepoints).

The figure shows the significant autoregressive effects (black solid lines) from internalizing symptoms to the next wave of internalizing symptoms (T+1), from externalizing symptoms to the next wave of externalizing symptoms (T+1) and from monthly alcohol use to the next wave of monthly alcohol use (T+1). Significant effects from negative alcohol expectancies onto the monthly alcohol use, internalizing and externalizing symptoms variables are included in black (dashed lines). Non-significant effects are presented in gray. All CRIs and covariates were adjusted for in the model simultaneously.

# References

1. Hamaker EL, Kuiper RM, Grasman RP (2015) A critique of the cross-lagged panel model. Psychological Methods 20:102-116

2. Hamaker EL, Mulder JD, van Ijzendoorn MH (2020) Description, prediction and causation: Methodological challenges of studying child and adolescent development. Developmental Cognitive Neuroscience 46:100867

3. Achenbach TM, Ivanova MY, Rescorla LA, Turner LV, Althoff RR (2016) Internalizing/Externalizing Problems: Review and Recommendations for Clinical and Research Applications. J Am Acad Child Adolesc Psychiatry 55:647-656

4. Orth U, Clark DA, Donnellan MB, Robins RW (2021) Testing prospective effects in longitudinal research: Comparing seven competing cross-lagged models. J Pers Soc Psychol 120:1013-1034

5. Usami S, Murayama K, Hamaker EL (2019) A unified framework of longitudinal models to examine reciprocal relations. Psychol Methods 24:637-657

6. Goodman R (2001) Psychometric properties of the strengths and difficulties questionnaire. J Am Acad Child Adolesc Psychiatry 40:1337-1345

7. Murray AL, Speyer LG, Hall HA, Valdebenito S, Hughes C (2022) A Longitudinal and Gender Invariance Analysis of the Strengths and Difficulties Questionnaire Across Ages 3, 5, 7, 11, 14, and 17 in a Large U.K.-Representative Sample. Assessment 29:1248-1261

8. Toseeb U, Oginni O, Rowe R, Patalay P (2022) Measurement invariance of the strengths and difficulties questionnaire across socioeconomic status and ethnicity from ages 3 to 17 years: A population cohort study. PLoS One 17:e0278385

9. Booth C, Moreno-Agostino D, Fitzsimons E (2023) Parent-adolescent informant discrepancy on the Strengths and Difficulties Questionnaire in the UK Millennium Cohort Study. Child and Adolescent Psychiatry and Mental Health 17:57

10. Rutter M, Tizard J, Whitmore K (1970) Education, health and behaviour. Longmans, London

11. Johnson J, Atkinson M, Rosenberg R (2015) Millennium Cohort Study Psychological, Developmental and Health Inventories. In:Centre for Longitudinal Studies

12. Kessler RC, Barker PR, Colpe LJ, Epstein JF, Gfroerer JC, Hiripi E, Howes MJ, Normand SL, Manderscheid RW, Walters EE, Zaslavsky AM (2003) Screening for serious mental illness in the general population. Archives of General Psychiatry 60:184-189

13. Pianta RC (1992) Child-Parent Relationship Scale.

14. Straus MA, Hamby SL (1997) Measuring physical and psychological maltreatment of children with the Conflict Tactics Scales. In: Out of darkness: Contemporary perspectives on family violence. Sage Publications, Inc, p 119-135

15. Rochebrochard EL (2012) Millennium Cohort Study Data Note 1: The home learning environment as measured at age 3. In:Centre for Longitudinal Studies

16. Guo J, Hawkins JD, Hill KG, Abbott RD (2001) Childhood and adolescent predictors of alcohol abuse and dependence in young adulthood. Journal of Studies on Alcohol 62:754-762

17. Maggs JL, Staff J, Patrick ME, Wray-Lake L, Schulenberg JE (2015) Alcohol use at the cusp of adolescence: a prospective national birth cohort study of prevalence and risk factors. The Journal of Adolescent Health 56:639-645

18. Class QA, Rickert ME, Larsson H, Lichtenstein P, D'Onofrio BM (2014) Fetal growth and psychiatric and socioeconomic problems: population-based sibling comparison. The British Journal of Psychiatry 205:355-361

19. Farooqi A, Hägglöf B, Sedin G, Gothefors L, Serenius F (2007) Mental health and social competencies of 10- to 12-year-old children born at 23 to 25 weeks of gestation in the 1990s: a Swedish national prospective follow-up study. Pediatrics 120:118-133

20. Arpi E, Ferrari F (2013) Preterm birth and behaviour problems in infants and preschool-age children: a review of the recent literature. Developmental Medicine and Child Neurology 55:788–796

21. Manzardo AM, Madarasz WV, Penick EC, Knop J, Mortensen EL, Sorensen HJ, Mahnken JD, Becker U, Nickel EJ, Gabrielli WF (2011) Effects of premature birth on the risk for alcoholism appear to be greater in males than females. Journal of Studies on Alcohol and Drugs 72:390–398

22. Day NL, Helsel A, Sonon K, Goldschmidt L (2013) The association between prenatal alcohol exposure and behavior at 22 years of age. Alcoholism, Clinical and Experimental Research 37:1171–1178

23. Duko B, Pereira G, Tait RJ, Bedaso A, Newnham J, Betts K, Alati R (2022) Prenatal alcohol exposure and offspring subsequent alcohol use: A systematic review. Drug and Alcohol Dependence 232:109324

24. Nomura Y, Gilman SE, Buka SL (2011) Maternal smoking during pregnancy and risk of alcohol use disorders among adult offspring. Journal of Studies on Alcohol and Drugs 72:199-209

25. Sutin AR, Flynn HA, Terracciano A (2017) Maternal cigarette smoking during pregnancy and the trajectory of externalizing and internalizing symptoms across childhood: Similarities and differences across parent, teacher, and self reports. Journal of Psychiatric Research 91:145-148

26. Rodriguez A (2010) Maternal pre-pregnancy obesity and risk for inattention and negative emotionality in children. Journal of Child Psychology and Psychiatry, and Allied Disciplines 51:134–143

27. Van Lieshout RJ, Schmidt LA, Robinson M, Niccols A, Boyle MH (2013) Maternal pre-pregnancy body mass index and offspring temperament and behavior at 1 and 2 years of age. Child Psychiatry and Human Development 44:382-390

28. Bandiera FC, Richardson AK, Lee DJ, He JP, Merikangas KR (2011) Secondhand smoke exposure and mental health among children and adolescents. Archives of Pediatrics & Adolescent Medicine 165:332-338

29. Luk TT, Wang MP, Suen YN, Koh DS, Lam TH, Chan SS (2018) Early childhood exposure to secondhand smoke and behavioural problems in preschoolers. Scientific Reports 8:15434

30. Brody GH, Ge X (2001) Linking parenting processes and self-regulation to psychological functioning and alcohol use during early adolescence. Journal of Family Psychology 15:82–94

31. Kingsbury M, Sucha E, Manion I, Gilman SE, Colman I (2020) Adolescent Mental Health Following Exposure to Positive and Harsh Parenting in Childhood. Can J Psychiatry 65:392-400

32. Nolan A, Smyth E (2021) Risk and protective factors for mental health and wellbeing in childhood and adolescence. In:The Economic and Social Research Institute (ESRI), Dublin

33. Yap MBH, Cheong TWK, Zaravinos-Tsakos F, Lubman DI, Jorm AF (2017) Modifiable parenting factors associated with adolescent alcohol misuse: a systematic review and meta-analysis of longitudinal studies. Addiction 112:1142–1162

34. Garriga A, Martínez-Lucena J, Moreno A (2019) Parents’ relationship quality and children’s externalizing problems: The moderating role of mother–child relations and family socio-demographic background. Child & Adolescent Social Work Journal 36:137-154

35. Visser L, De Winter AF, Reijneveld SA (2012) The parent–child relationship and adolescent alcohol use: a systematic review of longitudinal studies. BMC Public Health 12:1-16

36. Herman KC, Cohen D, Owens S, Latimore T, Reinke WM, Burrell L, McFarlane E, Duggan A (2016) Language Delays and Child Depressive Symptoms: the Role of Early Stimulation in the Home. Prevention Science 17:533–543

37. Liang Y, Cao H, Zhou N, Li J, Zhang L (2020) Early home learning environment predicts early adolescents' adjustment through cognitive abilities in middle childhood. Journal of Family Psychology 34:905–917

38. Kuppens S, Moore SC, Gross V, Lowthian E, Siddaway AP (2020) The Enduring Effects of Parental Alcohol, Tobacco, and Drug Use on Child Well-being: A Multilevel Meta-Analysis. Development and Psychopathology 32:765-778

39. Rossow I, Keating P, Felix L, McCambridge J (2016) Does parental drinking influence children's drinking? A systematic review of prospective cohort studies. Addiction 111:204-217

40. Anderson AS, Siciliano RE, Pillai A, Jiang W, Compas BE (2023) Parental drug use disorders and youth psychopathology: Meta-analytic review. Drug and Alcohol Dependence 244:109793

41. McGovern R, Bogowicz P, Meader N, Kaner E, Alderson H, Craig D, Geijer-Simpson E, Jackson K, Muir C, Salonen D, Smart D, Newham JJ (2023) The association between maternal and paternal substance use and child substance use, internalizing and externalizing problems: a systematic review and meta-analysis. Addiction 118:804-818

42. Keyes M, Legrand LN, Iacono WG, McGue M (2008) Parental smoking and adolescent problem behavior: an adoption study of general and specific effects. The American Journal of Psychiatry 165:1338–1344

43. Mahabee-Gittens EM, Yolton K, Merianos AL (2021) Prevalence of Mental Health and Neurodevelopmental Conditions in US Children with Tobacco Smoke Exposure. Journal of Pediatric Health Care 35:32-41

44. Evans SE, Davies C, DiLillo D (2008) Exposure to domestic violence: A meta-analysis of child and adolescent outcomes. Aggression and Violent Behavior 13:131-140

45. Meyers JL, Sartor CE, Werner KB, Koenen KC, Grant BF, Hasin D (2018) Childhood interpersonal violence and adult alcohol, cannabis, and tobacco use disorders: variation by race/ethnicity? Psychological Medicine 48:1540-1550

46. Ivanova MY, Achenbach TM, Turner LV (2022) Associations of Parental Depression with Children's Internalizing and Externalizing Problems: Meta-Analyses of Cross-Sectional and Longitudinal Effects. Journal of Clinical Child and Adolescent Psychology 51:827-849

47. Kelly LM, Becker SJ, Wolff JC, Graves H, Spirito A (2017) Interactive Effect of Parent and Adolescent Psychiatric Symptoms on Substance Use among Adolescents in Community Treatment. Community Mental Health Journal 53:383-393

48. Hazell M, Thornton E, Haghparast-Bidgoli H, Patalay P (2022) Socio-economic inequalities in adolescent mental health in the UK: multiple socio-economic indicators and reporter effects. SSM - Mental Health 2:100176

49. Moore GF, Littlecott HJ (2015) School- and family-level socioeconomic status and health behaviors: multilevel analysis of a national survey in wales, United Kingdom. The Journal of School Health 85:267-275

50. Barr PB, Silberg J, Dick DM, Maes HH (2018) Childhood socioeconomic status and longitudinal patterns of alcohol problems: Variation across etiological pathways in genetic risk. Social Science & Medicine 209:51-58

51. Meyrose AK, Klasen F, Otto C, Gniewosz G, Lampert T, Ravens-Sieberer U (2018) Benefits of maternal education for mental health trajectories across childhood and adolescence. Social Science & Medicine 202:170–178

52. Poonawalla IB, Kendzor DE, Owen MT, Caughy MO (2014) Family income trajectory during childhood is associated with adolescent cigarette smoking and alcohol use. Addictive Behaviors 39:1383-1388

53. Sareen J, Afifi TO, McMillan KA, Asmundson GJ (2011) Relationship between household income and mental disorders: findings from a population-based longitudinal study. Archives of General Psychiatry 68:419-427

54. Fitzsimons E, Haselden L, Smith K, Gilbert E, Calderwood L, AgaliotiSgompou V, Veeravalli S, Silverwood R, Ploubidis G (2020) Millennium Cohort Study Age 17 Sweep (MCS7): User Guide. In:UCL Centre for Longitudinal Studies. , London
